# A case of primary malignant melanoma of the esophagogastric junction with abscopal effect after nivolumab administration

**DOI:** 10.1186/s40792-021-01336-y

**Published:** 2021-12-09

**Authors:** Takahisa Yamaguchi, Sachio Fushida, Jun Kinoshita, Hiroto Saito, Mari Shimada, Shiro Terai, Hideki Moriyama, Koichi Okamoto, Keishi Nakamura, Itasu Ninomiya, Noriyuki Inaki

**Affiliations:** Department of Gastrointestinal Surgery, Kanazawa University Hospital, 13-1 Takara-machi, Kanazawa, Ishikawa 920-8641 Japan

**Keywords:** Melanoma, Esophagogastric junction, Abscopal effect

## Abstract

**Background:**

The abscopal effect is a rare phenomenon in which local irradiation causes tumor regression outside the irradiated area. There have been no reports of abscopal effect in patients with gastrointestinal melanoma with metastasis. Here, we report a case of primary malignant melanoma of the esophagogastric junction with abscopal effect after long-term treatment with nivolumab.

**Case presentation:**

A 75-year-old woman was referred to our hospital with a gastroesophageal lesion. Upper gastrointestinal endoscopy revealed a raised lesion on the posterior wall of the greater curvature of the cardia and tenderness in the lower esophagus. Immunostaining of the tumor biopsy showed positive staining for Melan-A, human melanoma black-45 (HMB45), and S-100, indicating malignant melanoma of the esophagogastric junction. Contrast-enhanced computed tomography (CT) of the abdomen showed a mildly stained lesion protruding into the cardiac part of stomach and enlarged surrounding lymph nodes. The patient was diagnosed with malignant melanoma of the esophagogastric junction and proximal gastrectomy with lower esophagus resection was performed. Histological examination showed large, round tumor cells with nuclear atypia. Immunostaining was positive for Melan A, HMB45, S-100 protein, and SRY-box transcription factor 10, and the final diagnosis was malignant melanoma of the esophagogastric junction, with regional lymph node metastases. Three months after surgery, follow-up CT indicated left pleural metastasis; therefore, the patient was administered nivolumab, an immune checkpoint inhibitor (ICI). Following three courses of nivolumab, the patient exhibited grade 3 renal dysfunction (Common Terminology Criteria for Adverse Events version 5.0). After that, we have not administered nivolumab treatment. Five months after the development of renal dysfunction, a CT scan demonstrated an unstained nodule within the pancreatic, and the patient was diagnosed with pancreatic metastasis; intensity-modulated radiotherapy was performed. Six months later, CT revealed pancreatic nodule and pleural metastasis was shrunk; after an additional 2 months, pleural metastasis and effusion had disappeared. The patient is alive with no additional lesions.

**Conclusions:**

We report a case of primary malignant melanoma of the esophagogastric junction with an abscopal effect following nivolumab treatment. The findings of this case report suggest that ICIs in combination with radiotherapy may be effective for treating metastatic or recurrent malignant melanoma of the gastrointestinal tract.

## Background

Malignant melanoma is a disease with poor prognosis that develops from melanocytes of the skin, oral cavity, nasal cavity, pharynx, uvea, and rectum. Primary melanoma of the gastrointestinal tract is rare [1–3], particularly in patients with melanoma esophagogastric junction of which there has been few case reports [4, 5].

The abscopal effect is a rare phenomenon in tumors, in which local radiotherapy of a tumor causes tumor regression outside of the irradiated area [6]. In recent years, it has been reported that the abscopal effect can be enhanced with the administration of immune checkpoint inhibitors (ICI) [7]; however, this area is still under-researched.

In this report, we report a case in which nivolumab was administered 5 months prior to irradiation, which resulted in an abscopal effect in the pleural metastasis.

## Case presentation

**Fig. 1:**
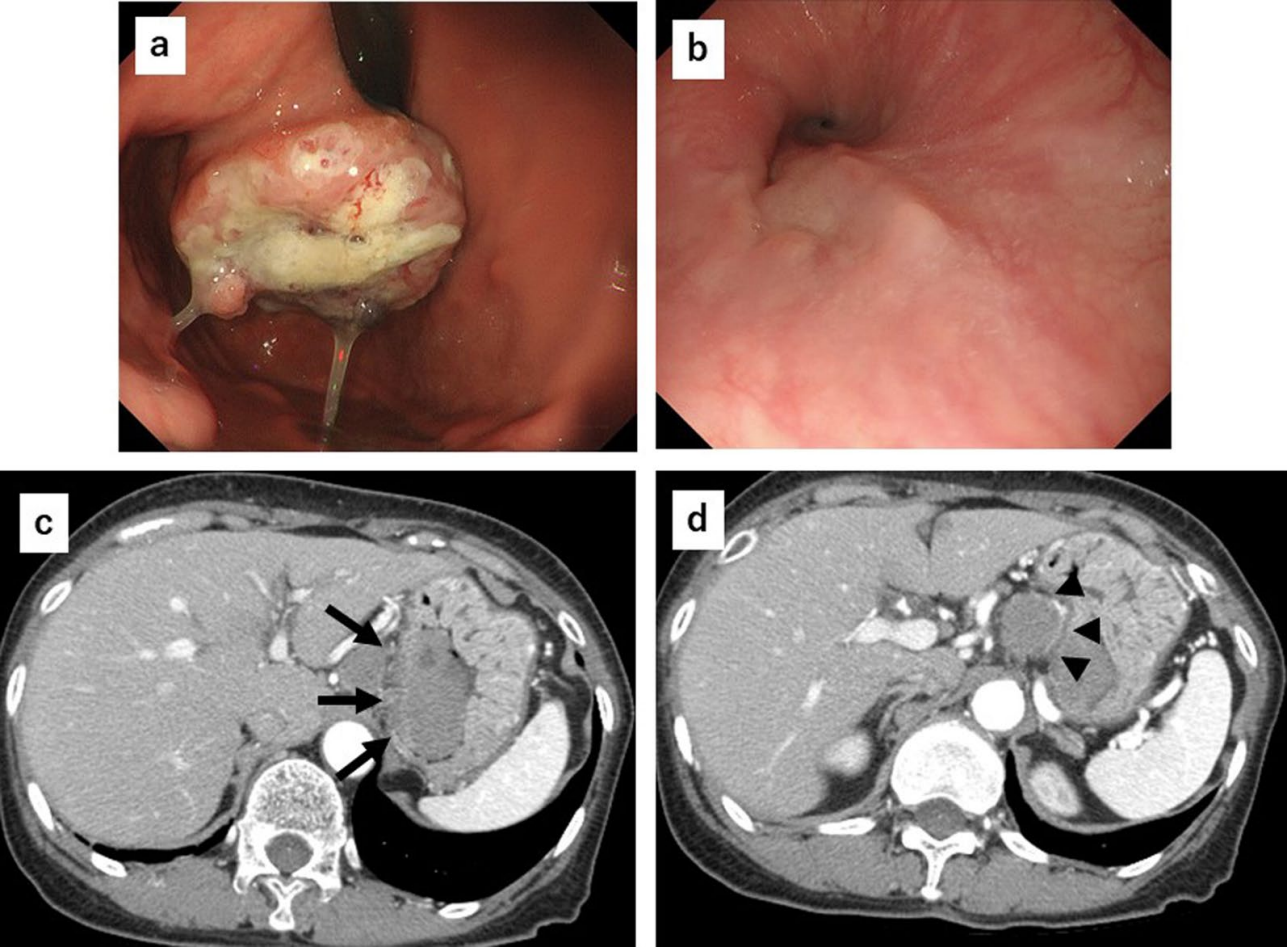
Preoperative findings. **a** Upper gastrointestinal endoscopy image of the raised lesion with ulceration on the posterior wall of the greater curvature of the cardia **b** Image of tumor invasion into the lower esophagus. **c** Contrast-enhanced CT of the abdomen showing a mildly stained lesion protruding into the cardiac part of stomach (black arrow). **d** Enlarged lymph node in the lesser curvature (black arrow)

**Fig. 2:**
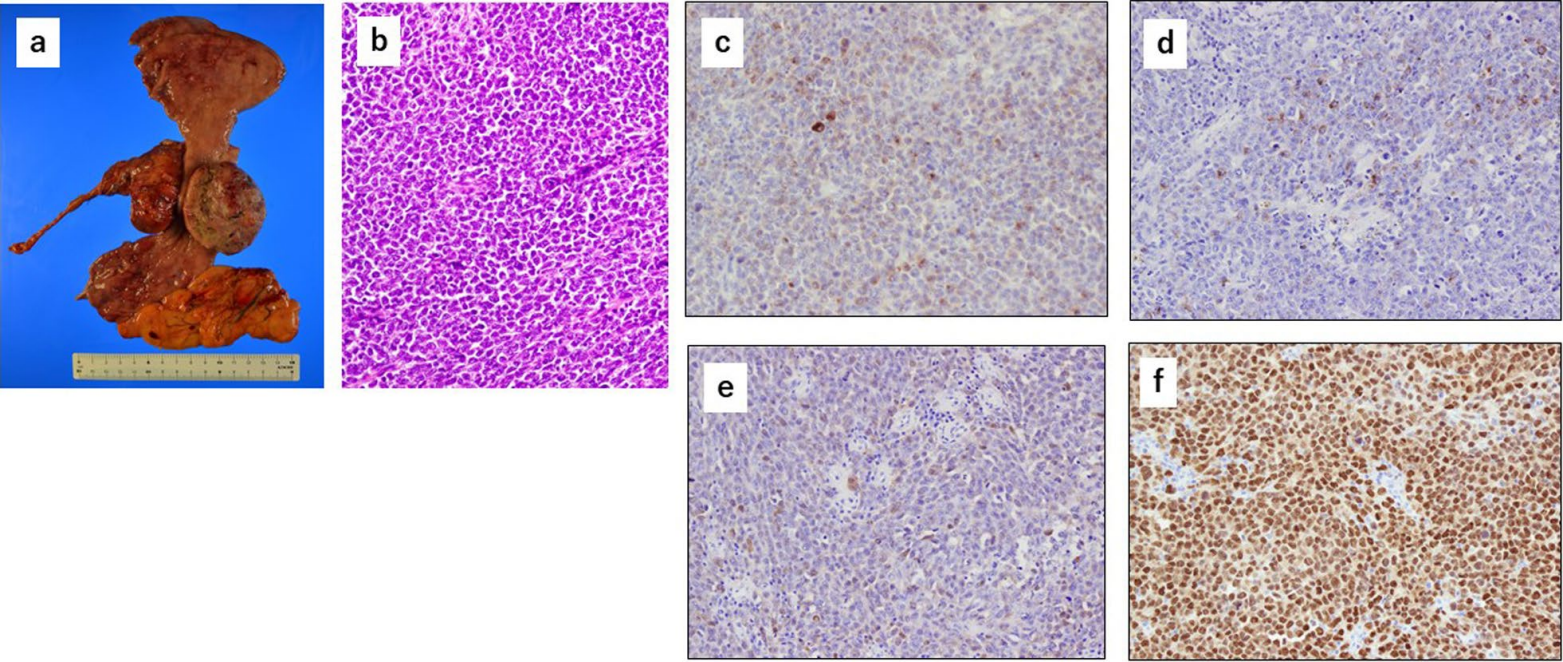
Pathological findings of the resected tumor. **a** Resected tissue of the esophagogastric junction. **b** Hematoxylin and eosin of the resected tumor. Immunostaining of **c** Melan A, **d** HMB45, **e** S-100 protein, and **f** SOX-10 in the resected tumor

**Fig. 3:**
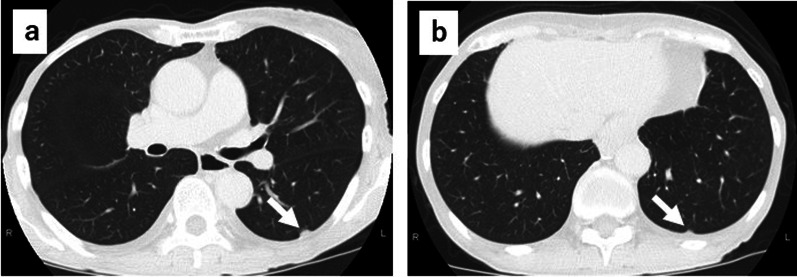
CT images of the chest. **a**, **b** CT of the chest, showing nodules in the subpleural area of the lower lobe of the left lung (white arrow)

**Fig. 4:**
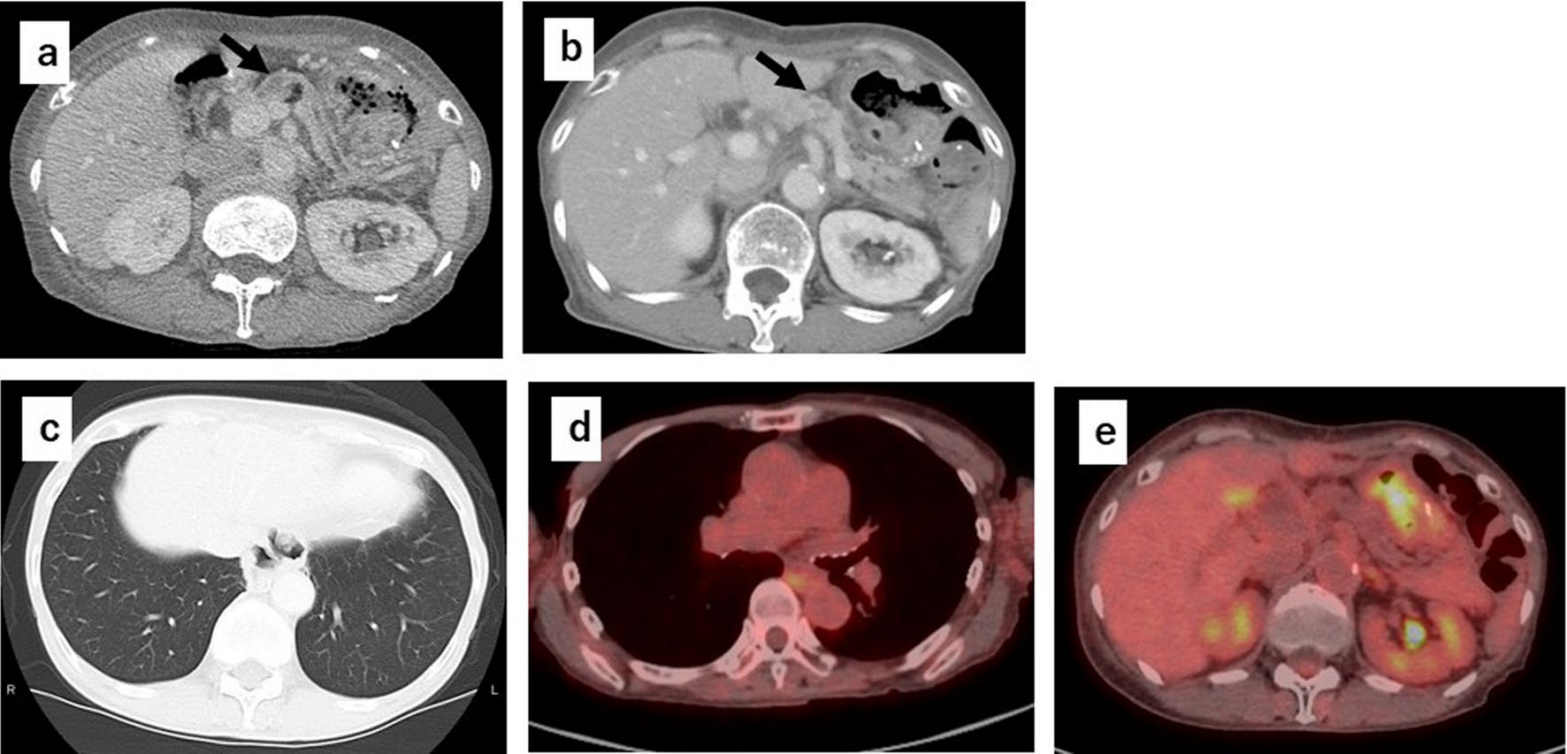
CT images of the abdominal area. **a** Abdominal CT showing unstained nodule in the pancreas (black arrow) and dilation of the caudal pancreatic duct. **b** CT indicating the obscured nodule in the pancreas (black arrow) after radiotherapy. **c** CT scan showing absence of metastatic lesions and subpleural and pleural effusion after radiotherapy. **d**, **e** PET–CT scan showing the absence of abnormal accumulation in the lung area and pancreas

**Fig. 5:**
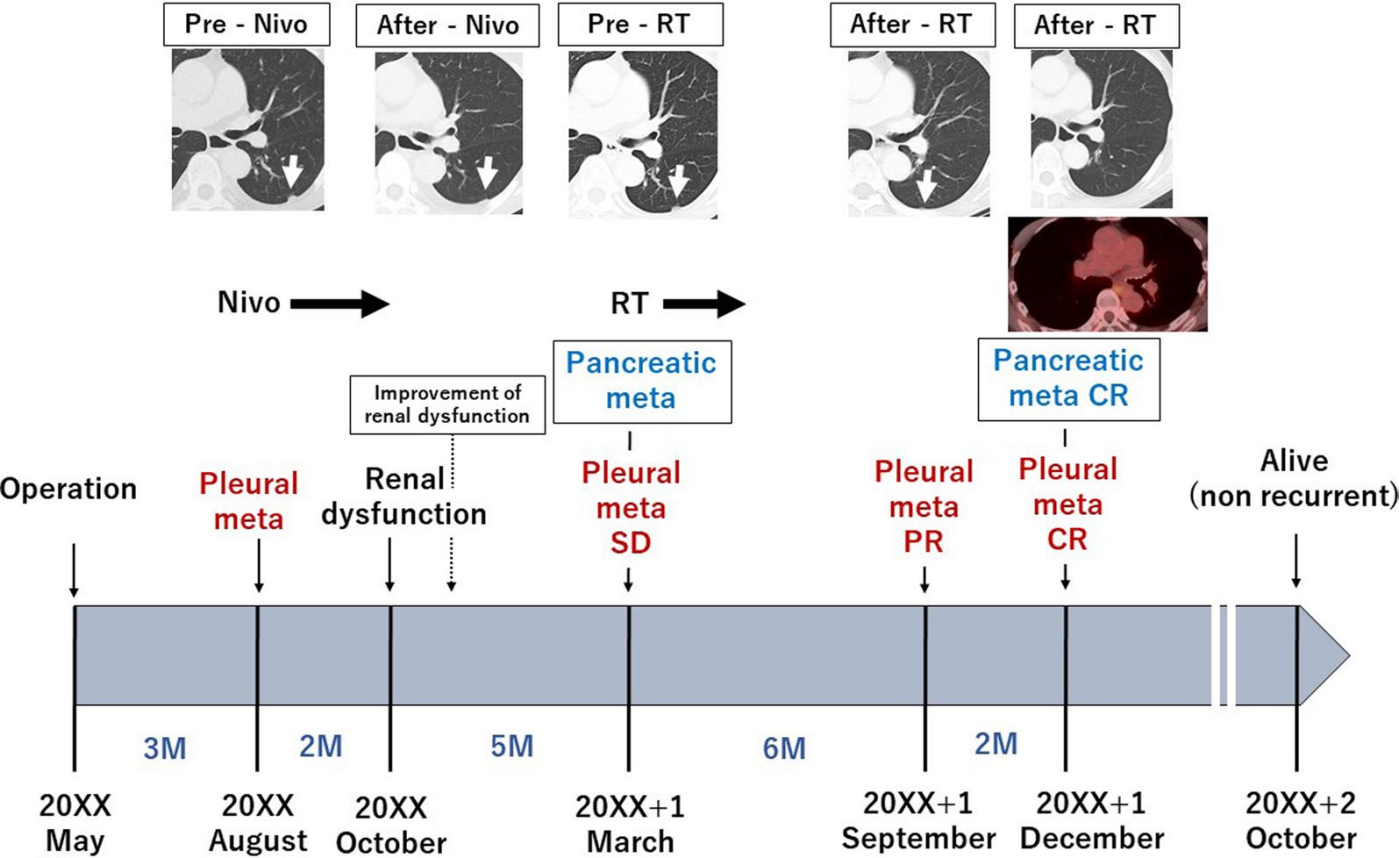
Timeline of the treatment interventions from surgery to metastasis and complete response to treatment. Pre: previous; RT: radiation therapy; Meta: metastasis; Nivo: nivolumab; PR: partial response; CR: complete response; SD: stable disease; M: months

A 75-year-old woman was referred to our hospital with a gastroesophageal lesion. Upper gastrointestinal endoscopy revealed a raised lesion with ulceration on the posterior wall of the greater curvature of the cardia. The endoscopy also indicated tenderness in the lower esophagus and tumor invasion was suspected (Fig. 1a, b). Immunostaining of the tumor biopsy showed positive staining for Melan-A, human melanoma black-45 (HMB45), and S-100 protein (+), indicating malignant melanoma of the esophagogastric junction. Contrast-enhanced computed tomography (CT) of the abdomen showed a mildly stained lesion protruding into the cardiac part of stomach and enlarged perigastric lymph nodes (the right paracardial lymph node and lesser curvature lymph node were lumped together) (Fig. 1c, d). No obvious distant metastasis was observed. Serum analysis indicated that squamous cell carcinoma antigen, carcinoembryonic antigen, and carbohydrate antigen 19–9 were within normal limits. A positron emission tomography (PET)– CT scan showed a high degree of fluorodeoxyglucose accumulation (maximum standardized uptake value: early = 13.2, delayed = 19.2) in the upper stomach and enlarged lymph nodes. Based on these findings, we performed proximal gastrectomy, lower esophagus resection, and double-tract reconstruction. We also performed resection of the enlarged lymph node in the lesser curvature of stomach and no obvious distant metastasis in the abdominal cavity was found. Histological examination of the resected tissue indicated an elastic, soft tumor that was located in the esophagogastric junction (6 × 5 cm, Fig. 2a). Analysis of the tumor cell morphology demonstrated large, round tumor cells with nuclear atypia and high mitotic activity (Fig. 2b). Immunostaining was positive for Melan-A, HMB45, S-100 protein and SRY-box transcription factor 10 (Fig. 2c–f), and the patient was diagnosed with malignant melanoma of the esophagogastric junction with regional lymph node metastases. Postoperative recovery was good, and the patient was discharged on 22 days post-operation. Three months after the surgery, a follow-up CT showed subpleural masses in the lower lobe of the left lung, with bilateral pleural effusion (Fig. 3a, b). As previous analyses of the primary tumor had revealed *BRAF*-wild-type, the patient was prescribed with nivolumab, human immunoglobulin G4 monoclonal antibody, and inhibitor of programmed death-1 (PD-1) in accordance with melanoma treatment guidelines. Following three courses of nivolumab treatment, the patient presented with grade 3 renal dysfunction (Common Terminology Criteria for Adverse Events version 5.0) [8]. The patient was prescribed steroid therapy for the immune-related adverse events that developed in response to the nivolumab treatment. Despite improved renal function, chemotherapy was discontinued at the patient's request. Five months after the presentation of renal dysfunction, a CT scan demonstrated an unstained nodule in the pancreas with dilation of the caudal pancreatic duct (Fig. 4a), although the size of the pleural metastasis was unchanged. Intensity-modulated radiotherapy (IMRT) was initiated for pancreatic metastasis treatment at 66 Gy. Six months after IMRT treatment, a CT scan revealed pancreatic nodule (Fig. 4b) and pleural metastasis was shrunk. Eight months after the IMRT (13 months after nivolumab treatment completion), the pleural mass and pleural effusion had disappeared (Fig. 4c). PET–CT showed no obvious abnormal accumulation (Fig. 4d, e). We hypothesized that the abscopal effect was caused by the radiation therapy, and further enhanced by the nivolumab treatment, which had finished 5 months earlier; the timeline of these events is shown in Fig. 5. Twelve months after the onset of the abscopal effect, no additional lesions were observed and the patient had discontinued all treatment.

## Discussion

In recent years, there have been few reports of an abscopal effect during the treatment of malignant melanoma [9, 10]. The abscopal effect was defined by Mole in 1953; this report described that localized irradiation at one site caused tumor regression at distant metastatic lesions [6]. At the time of this publication, the abscopal effect was considered a rare phenomenon in patients treated with radiation therapy alone. However, in recent years, it has been suggested that the combination of radiotherapy with chemotherapy is more likely to produce an abscopal effect. Previous findings suggest that the abscopal effect is in part caused by an irradiation-dependent induction of apoptosis, leading to production of damage-associated molecular pattern molecules (DAMPs), including high-mobility group box 1 (HMGB1) [11]. DAMP-associated apoptosis has also been termed immunogenic cell death (ICD). DAMPs activate dendritic cells by binding to toll-like receptors, which induces T lymphocyte activation [12, 13]. Conversely, radiation induces the expression of PD-L1 on the cell surface of tumor cells, which suppresses anti-tumor immunity [14]. Anti-PD-1 ICIs besides nivolumab have also shown to enhance the abscopal effect in several case reports [9, 15, 16]. For instance, cytotoxic T lymphocyte-associated protein 4 (CTLA-4) immunotherapy may also promote the abscopal effect [9].

In our case, nivolumab monotherapy was insufficient to reduce pleural metastasis, despite a previously reported complete response (CR) rate of 7.6–17.6% for nivolumab monotherapy in melanoma patients [17, 18]. The indication that the pleural metastasis had shrunk following irradiation of the pancreatic mass without additional chemotherapy, suggested that an abscopal effect had occurred. This finding was even more remarkable as the radiotherapy was administered 5 months after the completion of nivolumab treatment. Osa et al. reported that detection of nivolumab and T lymphocyte binding remained up to 20 weeks after the last nivolumab infusion, regardless of the total number of infusions [19]. This suggests that nivolumab has long-term efficacy and that in combination with irradiation, this likely caused the metastatic lesion regression and the abscopal effect. The onset of the abscopal effect has been observed as early as during irradiation; however, the onset is generally considered to occur within 12 months of beginning radiation therapy [20]; one report indicated an abscopal effect duration longer than 54 months [21]. This case had confirmed CR for 12 months following repeated PET–CT. This suggests that the neo-antigen originated from activated cytotoxic T cells (CTLs) at the pancreatic tumor via dendritic cells, resulting in the maintenance of ICD for the pleural tumor as well as the pancreatic tumor. Since irradiation is known to induce PD-L1 expression on the surface of tumor cells, patients with pancreatic tumors may not show CR. However, the long-term effects of nivolumab may have rescued the CTL activity against residual pancreatic tumors in the present case.

The primary treatment for patients with malignant melanoma is tumor resection; however, chemotherapy is recommended for patients with unresectable or recurrent malignant melanoma. In cases presenting with *BRAF* mutations, the first choice of treatment is a combination of the BRAF inhibitor, vemurafenib, and the mitogen-activated protein kinase inhibitor, cobimetinib [22, 23]. However, in recent years, the manipulation of immune checkpoints has provided a significant breakthrough in oncological treatment. Therefore, the combination of two ICIs (such as anti-PD1 and anti-CTLA4) has been considered as an experimental approach in clinical trials [24, 25]. In the present case, nivolumab was administered for pleural metastasis in the early postoperative period due to the absence of the *BRAF* mutation in the resected primary lesion. Furthermore, to our knowledge, the observation of the abscopal effect in a patient with gastrointestinal melanoma with metastasis has not been previously reported. Although the abscopal effect in metastatic cutaneous melanoma has been reported in several cases [26–28], the effect could be enhanced by co-administration of ICI therapy, such as nivolumab. The combination of radiation and ICI therapy may be sufficient to induce neo-antigen production; however, it is important to ensure that ICI treatments remain effective over several months.

## Conclusions

We presented a rare case of primary malignant melanoma of the esophagogastric junction that showed an abscopal effect against pleural metastasis as a response to long-acting ICI. The findings of this case suggest the potential of irradiation followed by ICI therapy as a beneficial treatment for melanoma of the gastrointestinal tract.

## Data Availability

All data generated during this study are included in this published article.

## Abbreviations

CT: Computed tomography
IMRT: Intensity-modulated radiotherapy
ICI: Immune checkpoint inhibitor
PD-1: Programmed death-1
DAMPs: Damage-associated molecular pattern molecules
HMGB1: High mobility group box1
ICD: Immunogenic cell death
CTLA-4: Cytotoxic T-lymphocyte-associated protein 4
